# Gastric cancer immune microenvironment score predicts neoadjuvant chemotherapy efficacy and prognosis

**DOI:** 10.1002/2056-4538.12378

**Published:** 2024-05-22

**Authors:** Shaoji Zhao, Yinan Liu, Li Ding, Chaoyue Zhang, Jinning Ye, Kaiyu Sun, Wu Song, Shirong Cai, Yulong He, Jianjun Peng, Jianbo Xu

**Affiliations:** ^1^ Department of Gastrointestinal Surgery The First Affiliated Hospital of Sun Yat‐Sen University Guangzhou PR China; ^2^ Department of Pathology The First Affiliated Hospital of Sun Yat‐Sen University Guangzhou PR China; ^3^ Digestive Diseases Center, Scientific Research Center The Seventh Affiliated Hospital of Sun Yat‐Sen University Shenzhen PR China

**Keywords:** gastric cancer, neoadjuvant chemotherapy, tumor immune microenvironment, tumor‐infiltrating immune cells, PD‐L1

## Abstract

The efficacy of neoadjuvant chemotherapy (NACT) in patients with advanced gastric cancer (GC) varies greatly. Thus, we aimed to verify the predictive value of tumor‐infiltrating immune cells (TIICs) on the treatment response to NACT and the prognosis of patients with advanced GC, and to explore the impact of NACT on the tumor immune microenvironment (TIME). Paired tumor tissues (pre‐ and post‐NACT) from patients with advanced GC were collected for this study. TIICs were assessed using immunohistochemistry staining and analyzed using logistic regression to establish an immune microenvironment score for GC (ISGC score) and predict NACT efficacy. Kaplan–Meier curves were used to evaluate the survival outcome of patients. The results showed that TIME was dramatically heterogeneous between NACT response and nonresponse patients. In the validation cohort, the ISGC score demonstrated good predictive performance for treatment response to NACT. Moreover, high ISGC indicated better long‐term survival in patients with advanced GC. Furthermore, tumor‐infiltrated T cells (CD3^+^ and CD8^+^) and CD11c^+^ macrophages were significantly increased in the response group, while CD163^+^ macrophages and FOXP3^+^ Treg cells were decreased after NACT. However, opposite results were exhibited in the nonresponse group. Finally, we found that the percentage of programmed cell death ligand 1 (PD‐L1)‐positive tumors was 31% (32/104) pre‐NACT and 49% (51/104) post‐NACT, and almost all patients with elevated PD‐L1 were in the NACT response group. The ISGC model accurately predicted NACT efficacy and classified patients with GC into different survival groups. NACT regulates the TIME in GC, which may provide strategies for personalized immunotherapy.

## Introduction

Gastric cancer (GC) is the fifth most common malignant tumor and the third most common cause of mortality worldwide [1]. Most patients with GC are diagnosed at advanced stages due to the lack of typical early symptoms. Despite advances in surgery and chemotherapy, the prognosis of patients with advanced GC remains poor [2]. Neoadjuvant chemotherapy (NACT) is the standard treatment for locally advanced GC to improve patient survival. However, the efficacy of NACT is approximately 50–60% in different clinical centers [3, 4, 5, 6]. Therefore, an accurate strategy to select patients who would benefit from NACT benefit is a critical issue in NACT clinical practice.

Nowadays, the efficacy of NACT has been evaluated using Response Evaluation Criteria in Solid Tumors (RECIST) guidelines. Given that CT/MRI images for treatment response judgment can only be acquired after NACT, the best opportunity for surgical resection may be missed. The tumor immune microenvironment (TIME) has recently emerged as a potential modulator of the response to chemotherapy and cancer progression. Tumor‐infiltrating lymphocytes (TILs) predict NACT response in triple‐negative breast cancer [7]. A large research cohort found that the tumor‐associated lymphocyte is an independent predictor of response to NACT in breast cancer [8]. The predictive value of tumor mutation burden, serum immune proteins, and RNAs from liquid biopsy on NACT response has also been reported in GC [9, 10, 11]. However, few studies have examined the association between infiltrating immune cells and the NACT response.

Tumor‐infiltrating immune cells influence cancer outcomes and are modulated by chemotherapy. Previous studies have reported that platinum agents increase tumor‐infiltrating dendritic cells and CD8^+^ T cells to induce immunogenic cell death [12, 13]. Mechanistically, increased tumor antigenicity and induction of tumor cell apoptosis recruit and reprogram the immune cells. However, evidence shows that chemotherapy increases programmed cell death ligand 1 (PD‐L1) expression and diminishes CD8^+^ T cells, which may contribute to tumor immune tolerance [14, 15]. However, the reprogramming of TIME is diverse due to chemotherapy sensitivity. In GC, natural killer (NK) cell recruitment decreases M1‐macrophage repolarization and increases effector T‐cell infiltration, associated with chemotherapy sensitivity [16, 17]. Treg decreases and CD8^+^ T cells elevate in NACT response patients [18]. However, the effect of NACT on the TIME and subsequent immunotherapy in patients with advanced GC remains uncertain.

New technologies such as multiplex immunohistochemistry, immunofluorescence, spatial biomarkers, and intelligent machine learning have advanced our understanding of the TIME. However, these methods require specialized platforms that are not widely available in clinical laboratories [19]. Therefore, in clinical practice, pathologists still rely on conventional immunohistochemistry to make diagnoses based on sequential analysis of individual biomarkers [20].

In this study, we aimed to construct a model based on the infiltrating immune cells to predict the efficacy of NACT. Additionally, we explored the correlation between NACT and the immune microenvironment to support subsequent immunotherapy selection.

## Materials and methods

### Patient population

**Figure 1:**
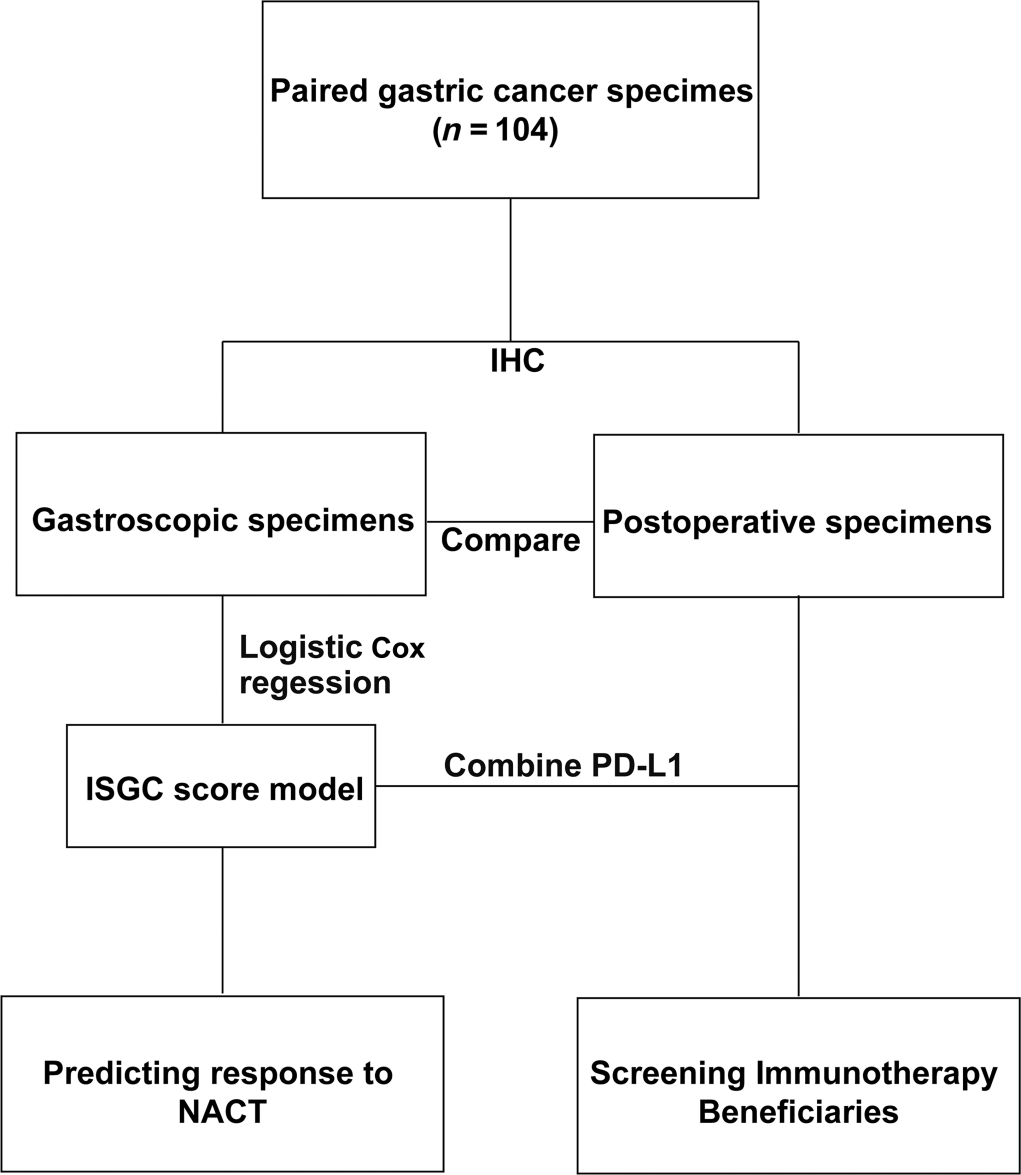
Flow chart of the study design. A total of 104 paired gastric cancer specimens before and after NACT were included. IHC, immunohistochemistry; NACT, neoadjuvant chemotherapy; PD‐L1, programmed cell death ligand 1.

Patients with locally advanced GC from the Gastrointestinal Surgery Center of the First Affiliated Hospital of Sun Yat‐Sen University from June 2011 to June 2020 were selected and individually reviewed by two researchers. Cases conformed to the following criteria: (1) age ≥18 years old; (2) histopathological diagnosis of gastric adenocarcinoma, and potentially resectable GC stage III, IV as determined by pretreatment contrast‐enhanced CT; (3) received and completed oxaliplatin combined with Teysuno NACT before R0 resection surgery, and without radiotherapy or other programs; (4) Eastern Cooperative Oncology Group score ≤1 point; (5) no bone marrow suppression (white blood cell count >3 × 10^9^/l, platelet count >100 × 10^9^/l). Cases were excluded if they had a history of other cancer, nontumor related death, or died within 1 month after surgery. Discrepancies of case inclusion were conferred by a principal investigator. The flowchart describing patient screening is shown in Figure 1.

Three consecutive cycles of oxaliplatin and Teysuno combination therapy were used for NACT for over 3 months. In each cycle, an intravenous infusion of oxaliplatin at a dose of 130 mg/m^2^ was administered on the first day, followed by oral administration of Teysuno twice daily based on the patient's body surface area (BSA < 1.25 m^2^, 80 mg/day; 1.25 m^2^ ≤ BSA < 1.50 m^2^, 100 mg/day; and BSA ≥ 1.50, 120 mg/day) for 14 days. The therapeutic response to NACT was evaluated using the RECIST (version 1.1) by two senior radiologists after three cycles. Overall survival (OS) was calculated based on follow‐up data collected every 3 months during the first 2 years after surgery and every 6 months from the third to fifth year. This study was conducted in accordance with the Declaration of Helsinki and was approved by the Ethics Committee of The First Affiliated Hospital of Sun Yat‐sen University, China [approval number: (2011) 59].

### Immunohistochemical (IHC) analysis

Formalin‐fixed and paraffin‐embedded blocks of gastroscopic biopsy specimens and postoperative specimens were cut into 4‐μm‐thick sections. The paraffin‐embedded sections were deparaffinized, rehydrated, subjected to antigen retrieval, and blocked for endogenous peroxidase activity. Next, the sections were incubated with anti‐CD3 (1:200 dilution, Abcam, Cambridge, UK), anti‐CD8 (1:200 dilution, Abcam), anti‐CD11c (1:150, Epitomics, Burlingame, CA, USA), anti‐CD163 (ab182422, diluted 1:500; Abcam), anti‐CD68 (ab955, diluted 1:300; Abcam), anti‐FOXP3 (1:150, Epitomics), and anti‐PD‐L1 (1:100, Epitomics) antibodies at 4 °C overnight. Then, the sections were incubated with horseradish peroxidase‐conjugated secondary antibody (G1214; Servicebio, Wuhan, Hubei, PR China) for DAB staining followed by hematoxylin counterstaining.

IHC results were evaluated by two independent observers. Tissue sections were screened using an inverted research microscope (model DM IRB; Leica, Wetzlar, Germany), and the three most representative fields were selected under a magnified view. The determination and quantification of positively stained cells were facilitated using QuPath v0.3.2. The visual field area map was imported into QuPath with the same resolution. The image type was set to brightfield (H‐DAB). The visual field area was divided using the Rectangle tool on the toolbar and the size of the rectangle was unified to 2,000 mm^2^. Next, ‘Estimate stain vectors’ was selected in Preprocessing on the Analyze column. The Min channel OD, Max total OD value, and Ignore extrema value were set to 0. In the Preprocessing column, select ‘Estimate stain vectors’. Set ‘Min channel OD’, ‘Max total OD’, and ‘Ignore extrema’ to 0.05%, 1%, and 1%, respectively. Check the ‘Exclude unrecognized colors’ option and click ‘Auto’ to complete the preliminary adjustment. To complete the initial adjustment, select ‘Positive cell detection’ in ‘Cell detection’. In the ‘Intensity threshold parameters’ interface, choose ‘Cytoplasm:DAB OD mean’ and define ‘Threshold 1+’, ‘2+’, and ‘3+’ as 0.1%, 0.2%, and 0.3%, respectively. Finally, derive the ‘Num Positive’ value on ‘Annotations’.

### Construction of immune microenvironment score for GC (ISGC) and validation

Immune cell number in the tumor‐enriched area was measured by pathologists by quantifying the number of T cells (CD3^+^ and CD8^+^), FOXP3^+^ Treg cells, and macrophages (CD68^+^, CD11c^+^, and CD163^+^). The number of positive cells was calculated in the same field (2,000 mm^2^). Immune cell number in the tumor‐enriched area was calculated as a percentage. The mean of the four quartiles was calculated and transformed into an immunoscore system (1, 2, 3, and 4 scores for each parameter). Immune score‐T cells (IS‐T) and immune score‐macrophages (IS‐M) were calculated by adding CD3^+^, CD8^+^, FOXP3^+^, CD68^+^, CD11c^+^, and CD163^+^ separately to avoid collinearity problems. In the training cohort, GC‐related meaningful indices were selected using univariate logistic regression analysis. Multiple logistic regression with significant indices was constructed. The ISGC of each sample was calculated using the coefficient of the immune scores (IS‐T and IS‐M) in multiple logistic regression.

The ISGC scores from the validation cohort were also calculated to verify the predictive value of ISGC for NACT efficacy. Receiver operating characteristic (ROC) curve analysis was performed to evaluate the predictive value of the ISGC score model. Decision curve analysis (DCA) was used to supervise the clinical benefits gained from the ISGC model.

### Statistical analysis

Statistical analysis was performed using Statistical Product and Service Solutions (SPSS) 22.0. Categorical variables were compared using chi‐square tests, with exact tests applied where appropriate. Continuous variables were compared using the Wilcoxon test with Pratt modification for paired samples. McNemar's test was used for categorical variables in paired samples. The median follow‐up was estimated using the Schemper method, while survival analysis was performed using the Kaplan–Meier (K–M) method. *p* ≤ 0.05 was considered significant.

## Results

### Patient characteristics and clinical outcomes

A cohort of 104 tumor tissue‐matched patients was analyzed before and after NACT. Among these patients, 62 were males (59.62%) and 42 were females (40.38%), with ages of 24–77 (54.48 ± 12.43) years. Before surgery, objective responses complete response (CR), partial response, stable disease (SD), and progressive disease (PD) were evaluated based on RECIST version 1.1 criteria (supplementary material, Figure S1A). Then, 46 (PR = 42 and CR = 4) and 58 (PD = 5 and SD = 53) patients were assigned to the response and nonresponse groups, respectively. All patients were randomized into the training (*n* = 74) or internal validation (*n* = 30) sets. Table 1 summarizes the patients' clinical characteristics. Only the number of metastasis‐positive lymph nodes (*p* = 0.045) differed significantly between the two cohorts. Other clinical features had no significant discrepancy between the two cohorts.

**Table 1:** Baseline characteristics of the gastric cancer patients included in the study

Variable	Total (*n* = 104)	Training cohort (*n* = 74)	Validation cohort (*n* = 30)	Statistic	*p*
Age, (years, mean ± SD)	54.48 ± 12.43	55.70 ± 12.34	51.47 ± 12.35	*t* = 1.586	0.116
Sex				*χ* ^2^ = 0.003	0.959
Female	42	30	12		
Male	62	44	18		
Preoperative T stage				*χ* ^2^ = 0.225	0.635
T2‐3	24	18	6		
T4	80	56	24		
Preoperative N status				*χ* ^2^ = 0.005	0.941
N0	9	7	2		
N+	95	67	28		
Preoperative M status				*χ* ^2^ = 0.050	0.823
M0	71	51	20		
M1	33	23	10		
Tumor location				*χ* ^2^ = 0.031	0.861
Esophagogastric junction	36	26	10		
Non‐esophagogastric junction	68	48	20		
Tumor size				*χ* ^2^ = 0.857	0.355
≤30 mm	49	37	12		
>30 mm	55	37	18		
Differentiation					
Well + moderately differentiated	58	41	17	*χ* ^2^ = 0.872	0.647
Poorly differentiated	36	27	9		
Unknown	10	6	4		
CA125 (IU/l)				*χ* ^2^ = 0.495	0.482
Normal	92	67	25		
Elevated	12	7	5		
CA199 (IU/l)				*χ* ^2^ = 1.070	0.301
Normal	86	63	23		
Elevated	18	11	7		
CEA (IU/l)				*χ* ^2^ = 0.499	0.480
Normal	71	49	22		
Elevated	33	25	8		
AFP (ng/ml)				*χ* ^2^ = 2.148	0.143
Normal	95	70	4		
Elevated	9	25	5		
Surgery type				*χ* ^2^ = 0.031	0.861
Laparoscopic	36	26	10		
Open surgery	68	48	20		
Resection type				*χ* ^2^ = 0.760	0.383
Part gastrectomy	35	23	12		
Total gastrectomy	69	51	18		
Total number of dissected lymph nodes	47.27 ± 19.02	48.20 ± 17.93	44.97 ± 21.64	*t* = 0.784	0.435
Number of positive lymph nodes	6.56 ± 11.74	5.09 ± 7.50	10.17 ± 18.14	*t* = −2.026	0.045
IS‐T score (mean ± SD)	2.50 ± 2.79	2.34 ± 2.76	2.90 ± 2.89	*t* = −0.929	0.355
IS‐M score (mean ± SD)	2.52 ± 2.53	2.46 ± 2.62	2.67 ± 2.32	*t* = −0.377	0.707

AFP, alpha‐fetoprotein; CA125, carbohydrate antigen 125; CA199, carbohydrate antigen 199; CEA, carcinoembryonic antigen.

### Immune microenvironment displays dissimilarities between NACT response and nonresponse patients

**Figure 2:**
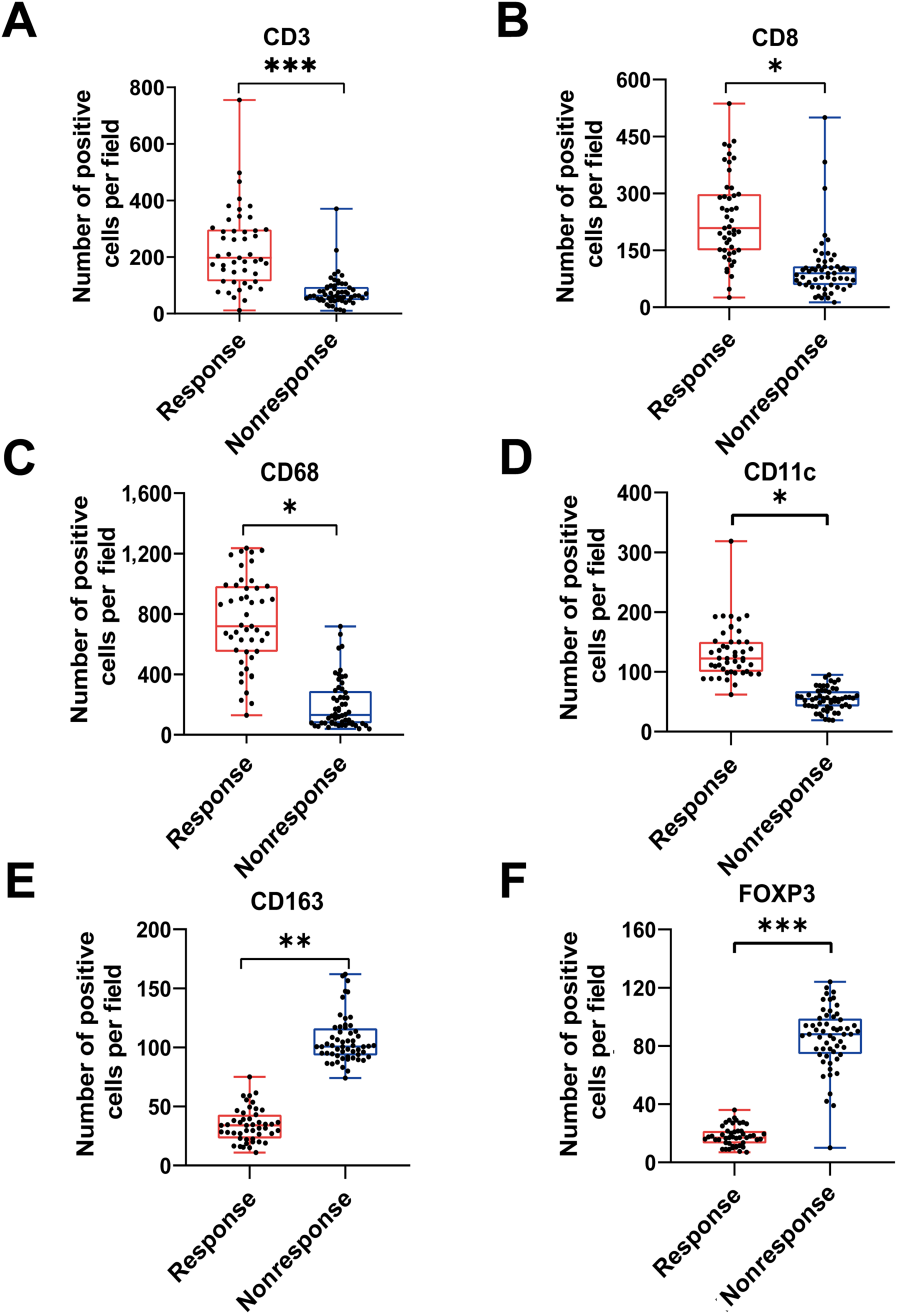
Variations in the infiltration density of six immune cells. (A) The quantification of CD3^+^ T cells in gastric specimens from the response and nonresponse groups before NACT. (B) The quantification of CD8^+^ T cells in gastric specimens from the response and nonresponse groups before NACT. (C) CD68^+^ macrophages in gastric specimens. (D) CD11c^+^ macrophages in gastric specimens. (E) The quantification of CD163^+^ macrophages. (F) The quantification of Foxp3^+^ Treg cells in gastric specimens. *P < 0.05; **P < 0.01; ***P < 0.001.

The IHC staining of CD3, CD8, FOXP3, CD68, CD11, and CD163 was performed on the preoperational gastroscopy samples from 104 patients before NACT. Quantification of these immune markers was performed using QuPath software, and the number of positive cells was counted (Figure S1B). The response group had significantly higher positive cells for CD3^+^, CD8^+^, CD68^+^, and CD11c^+^ than the nonresponse group but significantly lower FOXP3^+^ and CD163^+^ cells (Figure 2 and supplementary material, Figure S2A,B). The results indicated more abundant infiltrating CD8^+^ T cells, M1 macrophages, and scarce Tregs and M2 macrophages. In short, we found differences in the immune microenvironment between the NACT response and nonresponse patients with advanced GC.

### GC immune microenvironment predicts the NACT efficiency

**Figure 3:**
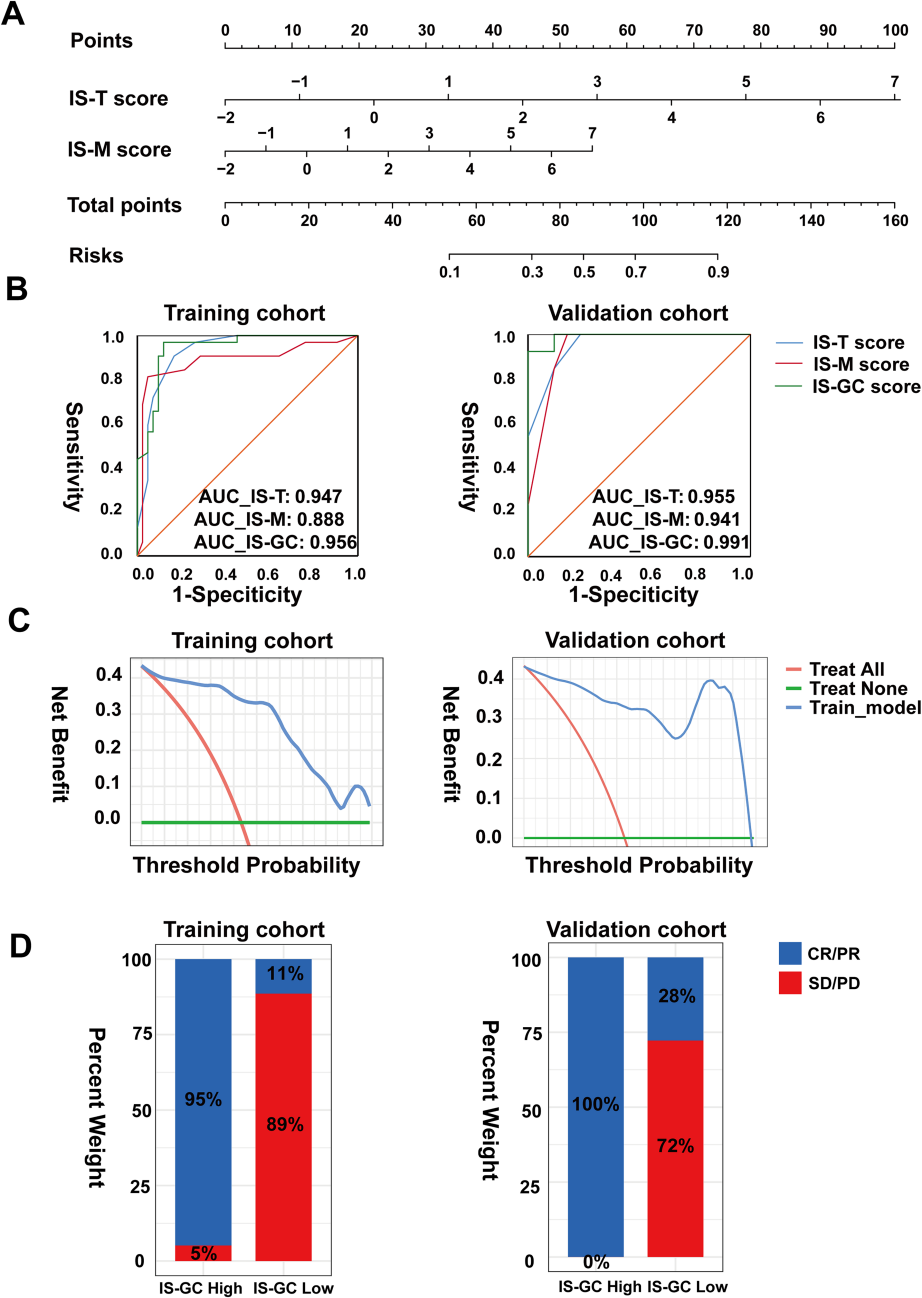
Development and validation of the ISGC score model. (A) Nomogram for predicting response to NACT. (B) ROC for the ISGC score in the training and validation cohorts. (C) DCA for the ISGC score in the training and validation cohorts. (D) The relationship between ISGC score and the efficacy of NACT. DCA, decision curve analysis; ROC, receiver operating characteristic curve.

Next, we investigated whether the immune microenvironment of GC could predict the efficacy of NACT. The statistically significant immune indicators and clinical characteristics were selected using univariate logistic regression analysis in the training cohort. The results showed that clinical features, such as the cTNM stage and the number of positive lymph nodes, were not statistically significant, whereas the IS‐T and IS‐M scores were statistically associated with NACT response (Table 2). Therefore, we used these two indicators to construct multiple logistic regression to predict the pathological response to NACT (IS‐T score odds ratio = 2.14, 95% CI: 1.37–3.35, *p* < 0.01; IS‐M score odds ratio = 1.52, 95% CI: 1.05–2.20, *p* = 0.027). The ISGC of each patient was calculated based on the coefficient of the logistic model using the following formula: ISGC = −3.511 + 0.761 × IS‐T score + 0.417 × IS‐M score. A nomogram was also generated for clinical practice (Figure 3A). ISGC was also calculated in the validation cohort. ROC and DCA curves were performed to estimate the accuracy and clinical benefit. The ROC curves showed that ISGC had a higher AUC than IS‐T and IS‐M scores in training and validation cohorts (Figure 3B). The DCA curves showed that the ISGC model had a great net benefit in NACT response prediction (Figure 3C). We also found that the proportion of NACT response patients was higher in the ISGC high group in the training and validation cohorts than in the ISGC low group (Figure 3D). These results suggested that ISGC had a great predictive performance in the pathological response to NACT efficiency in patients with GC.

**Table 2:** Characteristics of patients in the training cohort and univariate analysis *p* values

Variables	Beta	SE	*Z*	*p*	OR (95% CI)
Age (years)	0.02	0.02	0.89	0.376	1.02 (0.98–1.06)
Sex					
Male	0.22	0.48	0.46	0.642	1.25 (0.49–3.20)
Female					
Preoperative T stage					
T2‐3	0.36	0.54	0.66	0.507	1.43 (0.49–4.17)
T4					
Preoperative N status					
N0	−0.02	0.80	−0.02	0.983	0.98 (0.20–4.74)
N+					
Preoperative M status					
M0	0.27	0.51	0.53	0.593	1.31 (0.49–3.52)
M1					
Tumor location					
Esophagogastric junction	−0.18	0.49	−0.37	0.710	0.83 (0.32–2.18)
Non‐esophagogastric junction					
Tumor size					
≤30 mm	0.67	0.48	1.40	0.161	1.95 (0.77–4.96)
>30 mm					
Differentiation					
Well + moderately differentiated	0.15	0.87	0.17	0.867	1.16 (0.21–6.43)
Poorly differentiated					
Unknown					
CA125 (IU/l)					
Normal	0.62	0.80	0.77	0.441	1.86 (0.38–8.96)
Elevated					
CA199 (IU/l)					
Normal	−0.34	0.68	−0.50	0.619	0.71 (0.19–2.69)
Elevated					
CEA (IU/l)					
Normal	0.54	0.50	1.08	0.279	1.71 (0.65–4.52)
Elevated					
AFP (ng/ml)					
Normal	−0.01	0.03	−0.35	0.728	0.99 (0.94–1.05)
Elevated					
Surgery type					
Laparoscopic	0.66	0.49	1.35	0.178	1.94 (0.74–5.12)
Open surgery					
Resection type					
Part gastrectomy	−0.25	0.51	−0.48	0.632	0.78 (0.29–2.13)
Total gastrectomy					
Total number of dissected lymph nodes	−0.00	0.01	−0.35	0.727	1.00 (0.97–1.02)
Number of positive lymph nodes	−0.02	0.03	−0.59	0.552	0.98 (0.92–1.05)
IS‐T score	0.99	0.22	4.58	<0.001	2.69 (1.76–4.10)
IS‐M score	0.79	0.17	4.71	<0.001	2.21 (1.59–3.07)

AFP, alpha‐fetoprotein; CA125, carbohydrate antigen 125; CA199, carbohydrate antigen 199; CEA, carcinoembryonic antigen.

### ISGC high indicates better OS in GC patients with NACT

**Figure 4:**
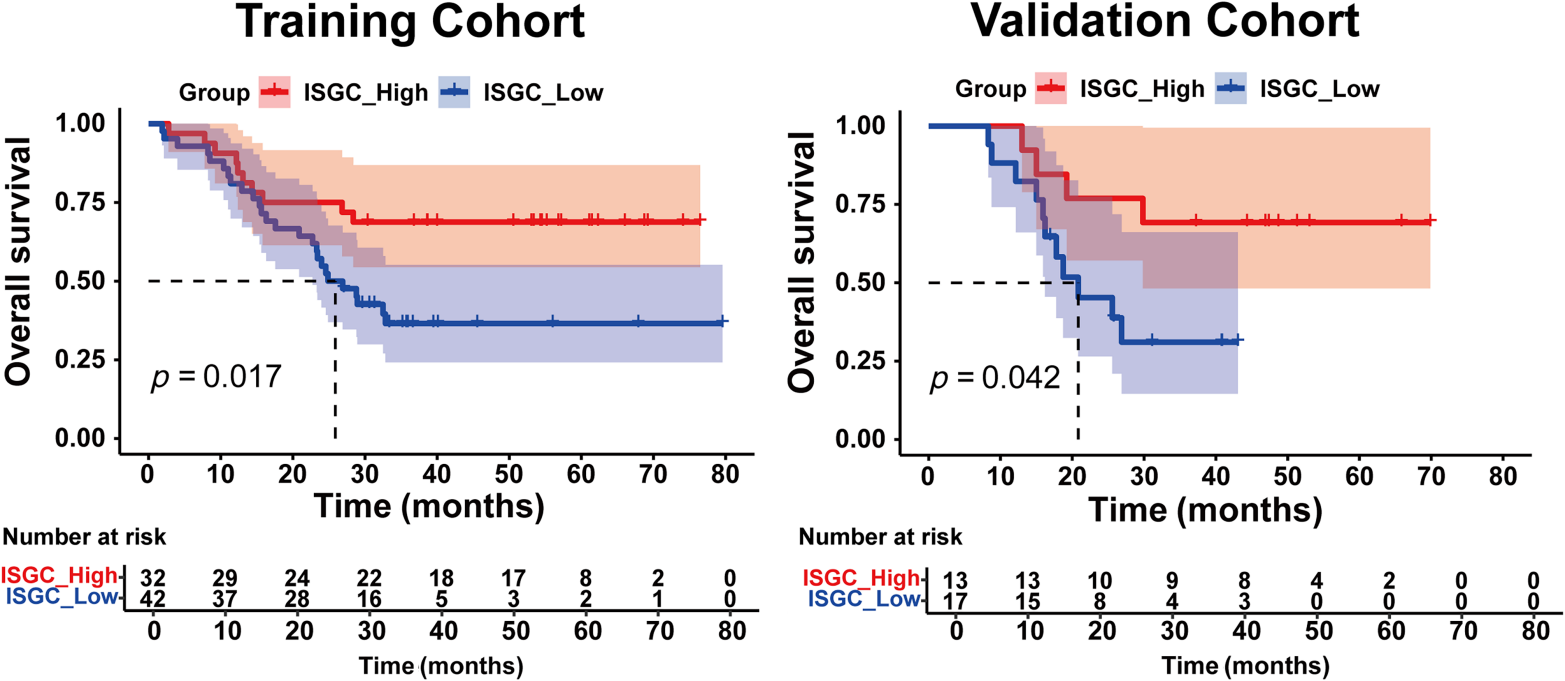
Comparison of survival between the ISGC high and low score groups. (A) K–M curve showing the overall survival of patients in ISGC high and low groups from the training set. (B) K–M curve of overall survival of patients in ISGC high and low groups in the validation set.

We focused on the correlation between the ISGC score and the long‐term survival of GC patients undergoing NACT treatment because TIME was associated with survival outcomes. We used K–M curves to detect whether patients in ISGC high and low groups had separate OS. The K–M curves exhibited that patients with high ISGC had a better OS outcome than those with low ISGC in training and validation cohorts (Figure 4).

### NACT changes the immune microenvironment of GC patients

**Figure 5:**
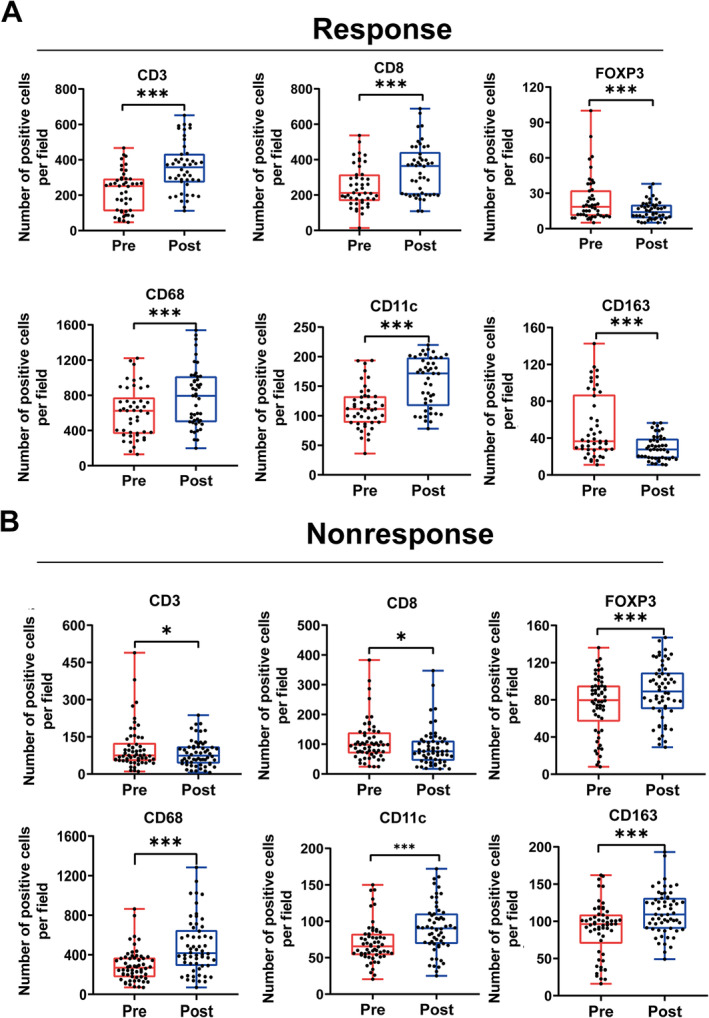
NACT alters the immune microenvironment in GC. (A) The infiltrating numbers of six immune cells before and after NACT evaluated in the response group. (B) The infiltrating numbers of six immune cells before and after NACT evaluated in the nonresponse group. *P < 0.05; **P < 0.01; ***P < 0.001.

After NACT, we conducted IHC staining and quantitative cytometric analysis on postoperative paired specimens from 104 patients using the same steps and modalities. The aim was to investigate changes in the immune microenvironment of GC. The results indicated a statistically significant increase in the number of tumor‐infiltrating T cells (CD3^+^, CD8^+^), CD68^+^ macrophages, and CD11c^+^ macrophages after NACT (supplementary material, Figure S3). In contrast, the quantity of Treg cells and CD163^+^ macrophages present in the immune microenvironment of GC exhibited a slight decrease following NACT, although this was not statistically significant (supplementary material, Figure S3). To investigate the correlation between the therapeutic effectiveness of NACT and the immune microenvironment of GC, we analyzed the immune cells in the response and nonresponse groups of NACT. We discovered that the number of T cells (CD3^+^, CD8^+^) in the response group increased significantly after NACT, while the number of Treg cells decreased (Figure 5A and supplementary material, Figure S4A). In contrast, the nonresponse group showed a statistically significant decrease in T cells and Treg cells (Figure 5B and supplementary material, Figure S4C). It is worth noting that the response group had the opposite situation to the nonresponse group. Our study found that the number of macrophages (CD68^+^, CD11c^+^) increased after NACT in both groups (supplementary material, Figures S3 and S4B,D). However, the response group showed a significantly greater increase than the nonresponse group. Additionally, the response group showed a decrease in the number of infiltrating CD163^+^ macrophages, while the nonresponse group showed an increase (Figure 5A,B and supplementary material, Figure S4B,D). These changes were statistically significant. In conclusion, NACT remodeled the immune microenvironment and the changes in the numbers of infiltrating immune cells correlated with the efficacy of NACT. This treatment activated anti‐tumor immunity in GC patients to a certain extent.

### ISGC score correlates with PD‐L1 expression in GC

**Figure 6:**
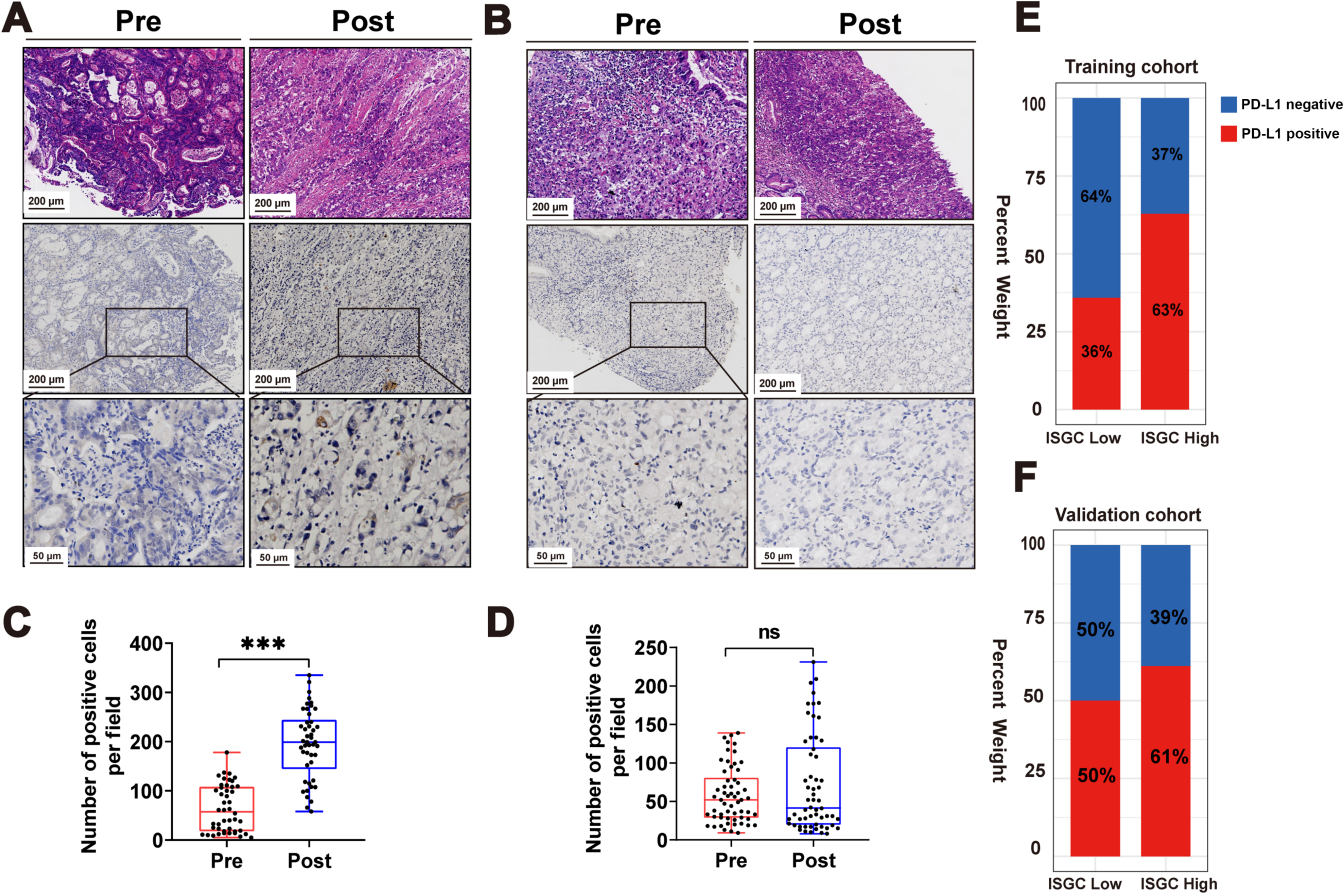
The correlation between ISGC score and changes in PD‐L1 expression. Changes in PD‐L1 expression in ISGC high score group (A) before and (C) after NACT. Changes in PD‐L1 expression in the ISGC low score group (B) before and (D) after NACT. Relationship between ISGC score and positivity rate of PD‐L1 expression after neoadjuvant chemotherapy in (E) the training and (F) the validation cohorts. ***P < 0.001; ns: *P* > 0.05.

The PD‐L1 expression level is a critical standard for immunotherapy selection in clinical practice. Therefore, we detected the change in PD‐L1 expression after NACT treatment. PD‐L1 expression dramatically increased after NACT treatment in the response groups (Figure 6A,C), while it hardly changed in the nonresponse groups (Figure 6B,D). Furthermore, after employing 5% as the cutoff point to define PD‐L1‐positivity, the proportion of PD‐L1‐positive patients was 31% (32/104) before NACT treatment and increased to 49% (51/104) after NACT. Moreover, the positivity rate increased from 54% (25/46) to 76% (35/46) in the response group, and from 12% (7/58) to 29% (16/58) in the nonresponse group. Relationship analysis between PD‐L1 and ISGC revealed that high ISGC correlated positively with the positive expression rate of PD‐L1. A higher probability of PD‐L1 positivity significantly correlated with a high ISGC score. We found that elevation of the PD‐L1 positivity rate after NACT treatment was mainly concentrated in the ISGC‐high group (Figure 6E,F). The ISGC score provides a reference for postoperative provision of combination immunotherapy.

## Discussion

The importance of NACT in advanced GC is increasing. In clinical practice, the cTNM staging system is a key decision‐making factor in selecting patients for NACT. However, patients with the same disease stage may experience different treatment effects. Recent publications have suggested that the efficacy of NACT for GC is approximately 30–50% [21]. Blindly administering NACT to all patients with advanced GC is not advisable and may be harmful. Factors affecting the efficacy of NACT for GC remain controversial. Therefore, it is critical to accurately select the population that benefits from NACT.

With a better understanding of the interaction between tumors and the immune microenvironment, an immunoscore based on the type, infiltration density, and location of immune cells in the tumor microenvironment has a strong predictive ability for colon cancer, even superior to the classic TNM system [22]. Moreover, its effectiveness has been confirmed in other solid tumors, such as breast and bladder cancers [23, 24]. Immunoscore is a robust prognostic factor and predictor of chemotherapy response [25]. The TIME and immunoscore are valuable predictors of malignancy and have been suggested as novel tools for tumor classification [26, 27, 28, 29]. One of the mechanisms of cytotoxic drugs is inducing immunogenic cell death of tumor cells, enhancing their immune induction effect [30]. Therefore, we should focus on the immune cells in the immune microenvironment to predict the efficacy of NACT in GC. Previous studies have also utilized the immune microenvironment to predict prognosis and screen for chemotherapy benefits in patients with GC [31]. Our research focused on using immune‐infiltrating cells in TIME to predict efficacy, a unique approach. Compared to traditional immune scoring methods, we focus on T cells and specifically study numerous tumor‐associated macrophages in the immune microenvironment. We constructed an ISGC score model containing six immune features using logistic regression. This model can accurately predict the efficacy of NACT and stratify patients to predict their prognoses.

With the arrival of the era of precision medicine, immunotherapy, especially the promotion of immune checkpoint inhibitors, such as PD‐1/PD‐L1 and CTLA‐4, has profoundly changed the pattern of solid tumor treatment [32]. Only a small number of patients respond to immunotherapy. The effectiveness of a single immune checkpoint inhibitor drug treatment is approximately 10–35% [33]. Therefore, identifying patients who will benefit, and improving treatment effectiveness, is a problem that must be solved in clinical practice. The CheckMate 649 study indicated that, in GC patients with CPS ≥ 5, the complete remission rate of chemotherapy combined with nivolumab was nearly twice that of chemotherapy alone [34]. The therapeutic effect of conventional cytotoxic drugs, such as oxaliplatin, is closely related to the immune microenvironment [35]. GC is a cold tumor; therefore, understanding the changes in the immune microenvironment before and after NACT may accelerate the development of synergistic combination immunotherapy to improve the clinical effectiveness of immune checkpoint inhibitors [36, 37]. According to the analysis based on the correlation between the score and PD‐L1, our model identified that the response group after NACT had a higher percentage of PD‐L1 positivity than the nonresponse group. Moreover, the increase in PD‐L1 positivity after NACT was mainly concentrated in patients who were ISGC‐high PD‐L1 negative before NACT. The model we developed is expected to become a new tool for guiding the precision treatment of GC.

However, although our study offers valuable insights, it is important to acknowledge its limitations as a single‐center retrospective study. The sample pool comprised specimens from the First Affiliated Hospital of Sun Yat‐Sen University in PR China, and most patients exhibited later TNM stages, which may hinder its generalizability. Additionally, our study only examined a limited selection of immune biomarkers, omitting relevant factors such as CTLA4, IL‐17, and MHC molecules. Therefore, multicenter, prospective clinical trials are necessary to validate our findings.

In conclusion, the ISGC score model is a highly effective tool to predict the response and prognosis of NACT, enabling the identification of patients with GC who may benefit from this treatment. Notably, based on ISGC and PD‐L1 expression, we found that patients with a high ISGC score and PD‐L1 negativity were more likely to benefit from postoperative combination immunotherapy. Consequently, ISGC may be useful for individualized adjuvant therapy and follow‐up.

## Author contributions statement

SZ and CZ were involved in data curation. YL, CZ and LD contributed to formal analysis. LD performed immunohistochemistry and evaluation of immunostaining. JY, KS and WS performed investigation. SC, YH and LD carried out methodology. YH, JP and JX were involved in project administration. JY, KS, WS, SC and YH were involved in providing resources. SZ and YL contributed to software and writing – original draft. JX was involved in supervision. WS, SC, YH, JP and JX performed validation. JX contributed to writing – review and editing.

## Supporting information


**Figure S1.** Determination of the effect of NACT and schematic illustration of positive immune cell counting
**Figure S2.** Representative images of six types of immune cell infiltration density in gastric endoscopic specimens from the NACT response and nonresponse groups
**Figure S3.** The infiltrating numbers of six immune cell types before and after NACT evaluated in all cases
**Figure S4.** Changes in T cells and macrophages in the response and nonresponse groups before and after NACT

## Data Availability

The datasets generated and/or analyzed during the current study are available from the corresponding author on reasonable request. Emails could be sent to the following address to obtain the shared data: xjianb@mail.sysu.edu.cn.
