# Supplementary material for: Gastric cancer immune microenvironment score predicts neoadjuvant chemotherapy efficacy and prognosis
Source: J Pathol Clin Res. 2024 May 22;10(3):e12378. doi: 10.1002/2056-4538.12378 (PMC11112142; doi:10.1002/2056-4538.12378)
Supplement: Supplementary file 1 — Figure S1. Determination of the effect of NACT and schematic illustration of positive immune cell counting Figure S2. Representative images of six types of immune cell infiltration density in gastric endoscopic specimens from the NACT response and nonresponse groups Figure S3. The infiltrating numbers of six immune cell types before and after NACT evaluated in all cases Figure S4. Changes in T cells and macrophages in the response and nonresponse groups before and after NACT [file CJP2-10-e12378-s001.pdf]

Gastric cancer immune microenvironment score predicts neoadjuvant chemotherapy efficacy and prognosis

S Zhao, Y Liu, L Ding *et al.* *J Pathol Clin Res* https://doi.org/10.1002/2056-4538.12378

Supplementary Figures S1–S4

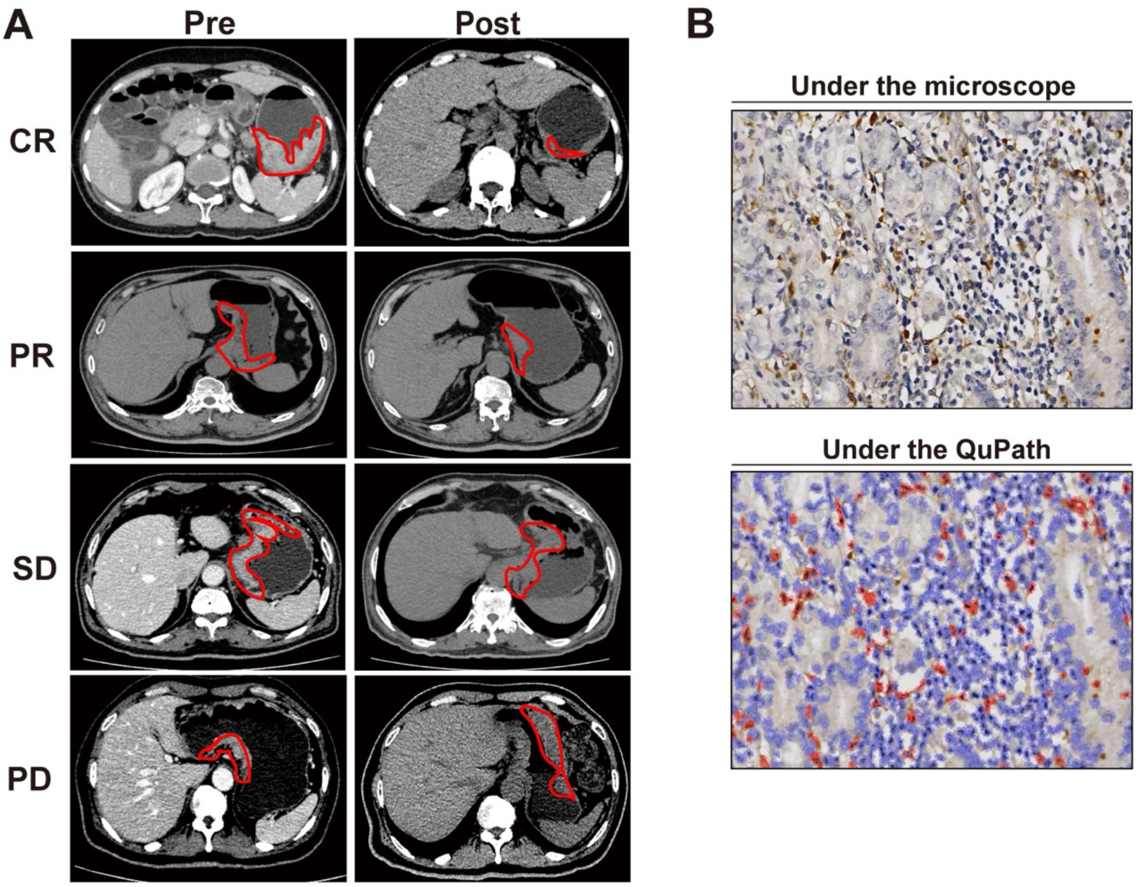

Figure S1. Determination of the effect of NACT and schematic illustration of positive immune cell counting. (A) Assessing NACT efficacy based on CT imaging and RECIST criteria. (B) Performing positive immune cell counting on IHC images using QuPath software. RECIST, response evaluation criteria in solid tumors.

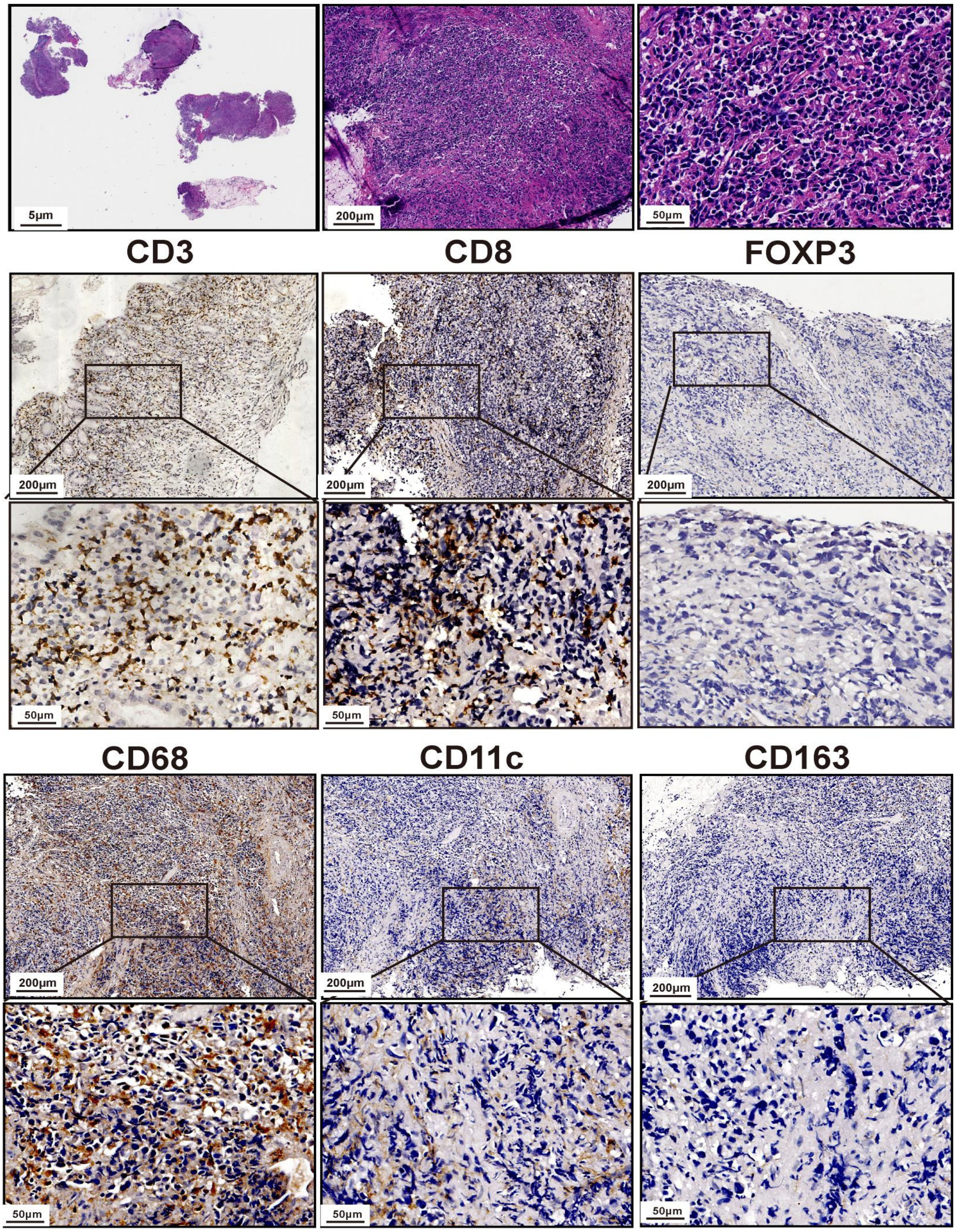

Figure S2A. Representative images of six types of immune cell infiltration density in gastric endoscopic specimens from the NACT response group.

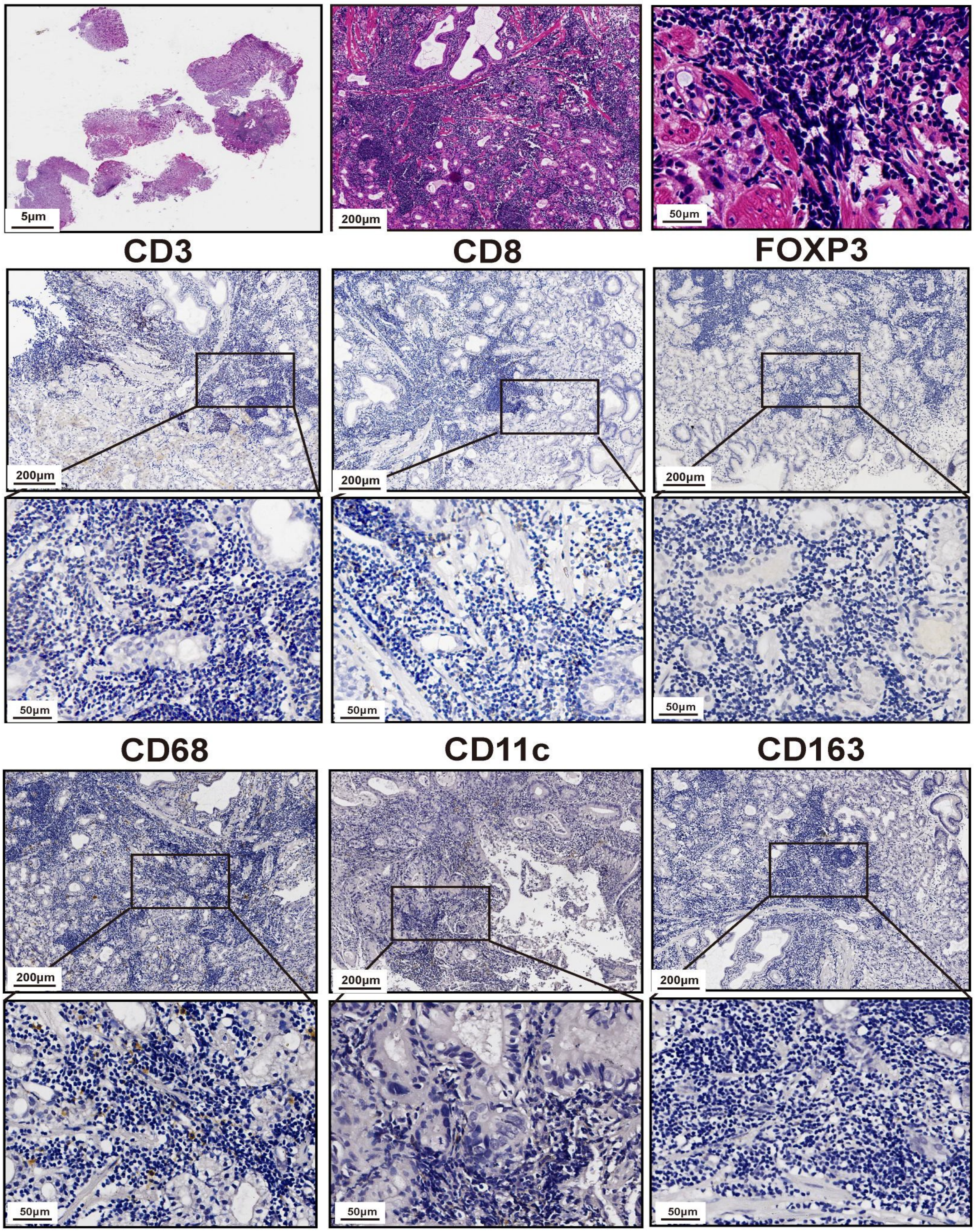

Figure S2B. Representative images of six types of immune cell infiltration density in gastric endoscopic specimens from the NACT non-response group.

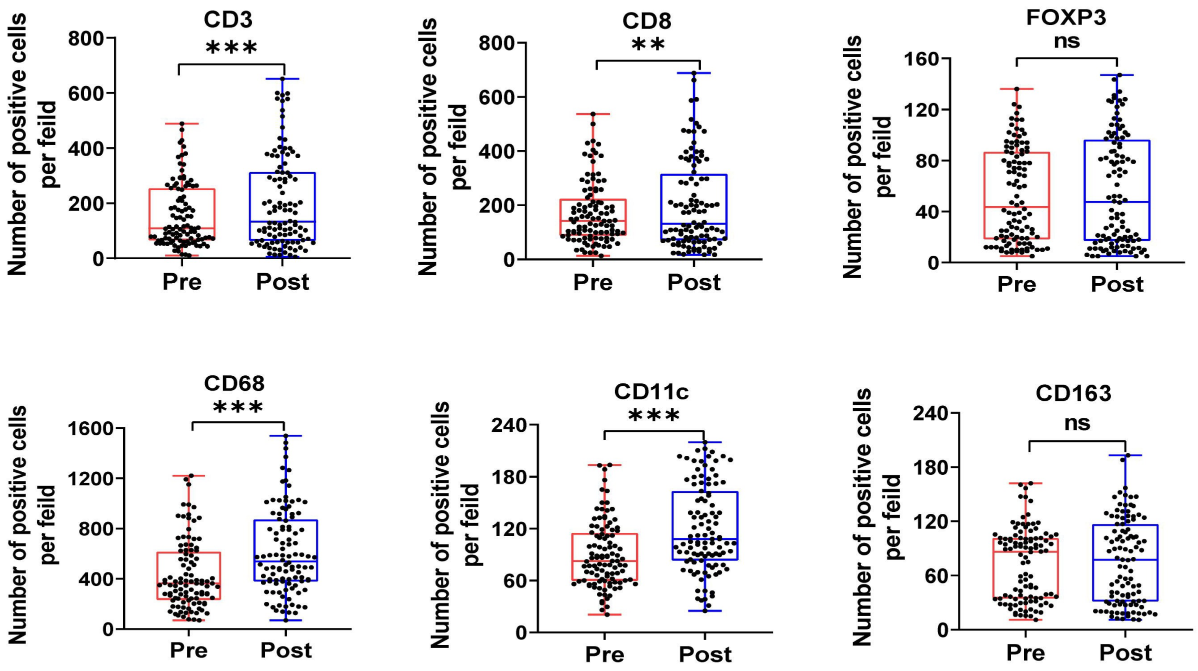

Figure S3. The infiltrating numbers of six immune cell types before and after NACT evaluated in all cases.

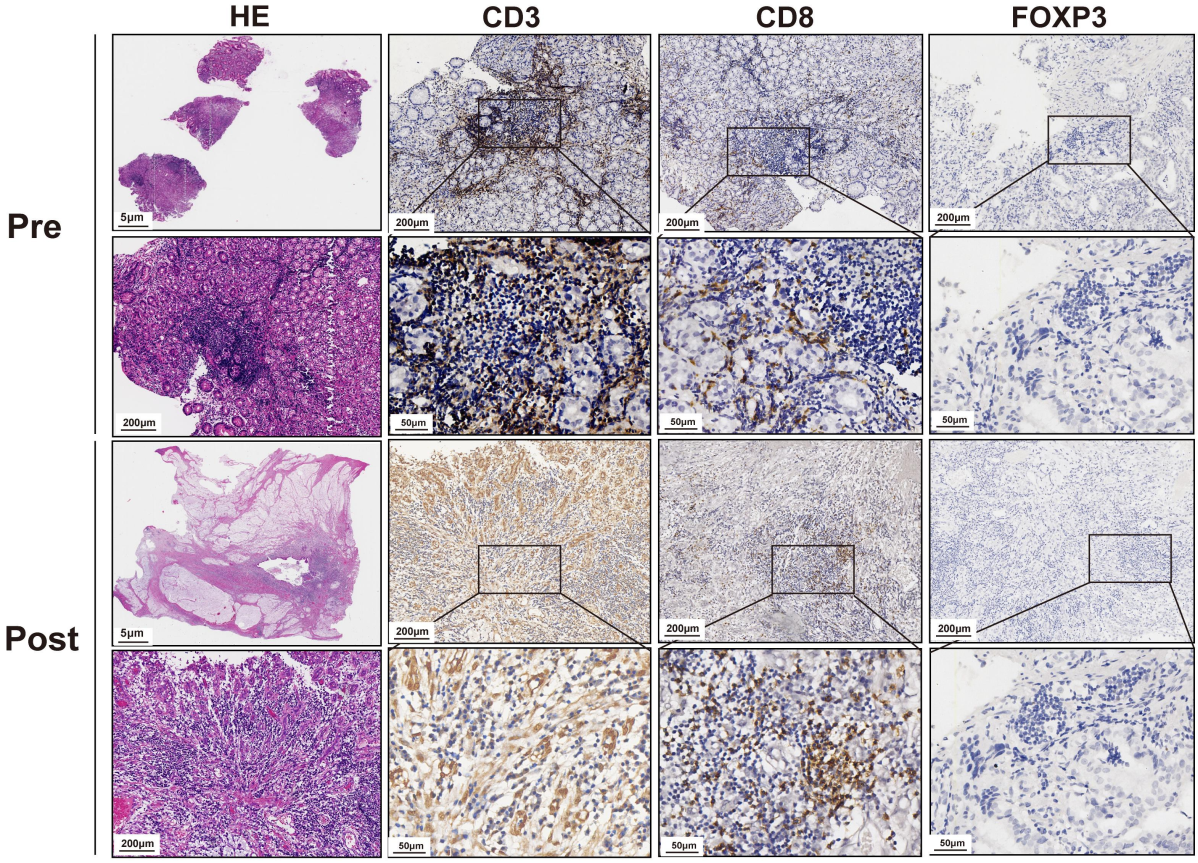

Figure S4A. Changes in T cells in the response group before and after NACT for GC.

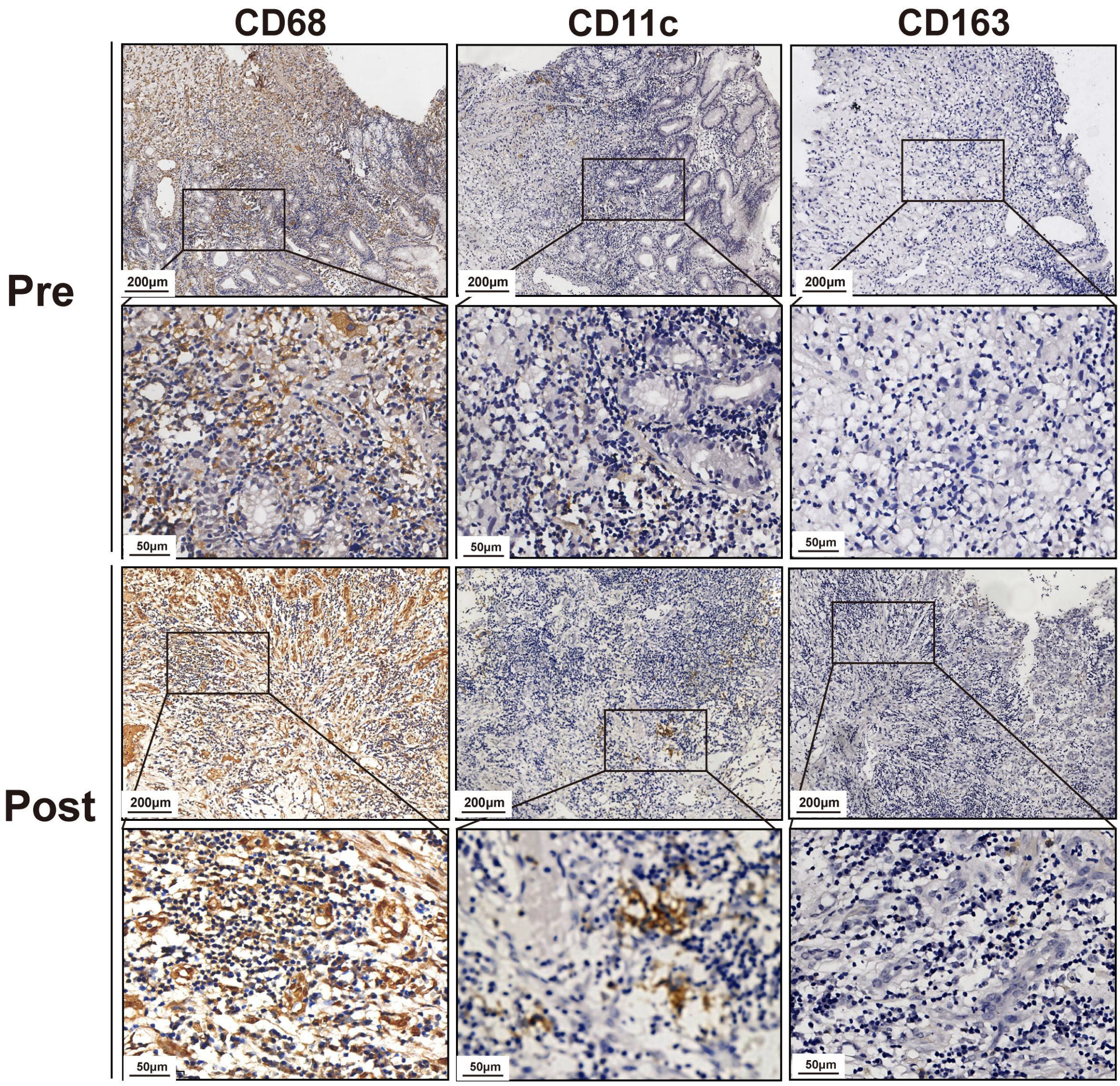

Figure S4B. Changes in macrophages in the response group before and after NACT for GC.

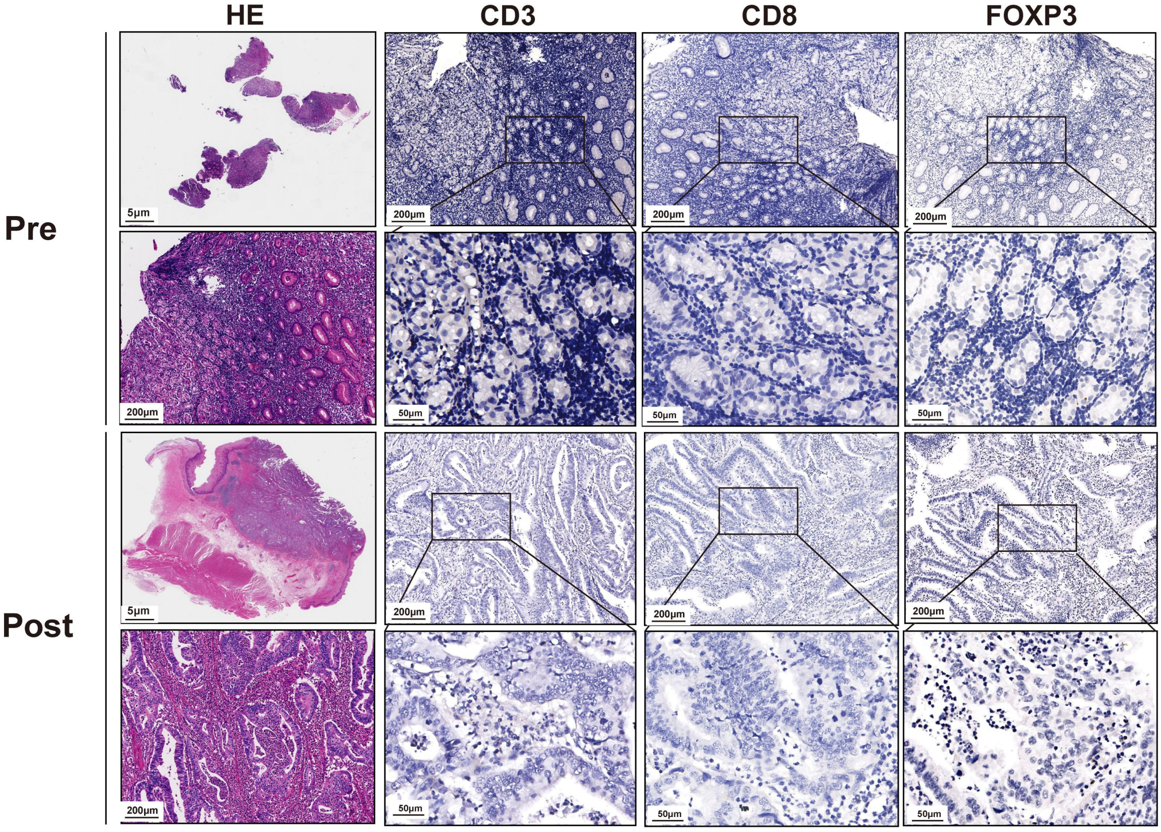

Figure S4C. Changes in T cells in the non-response group before and after NACT for GC.

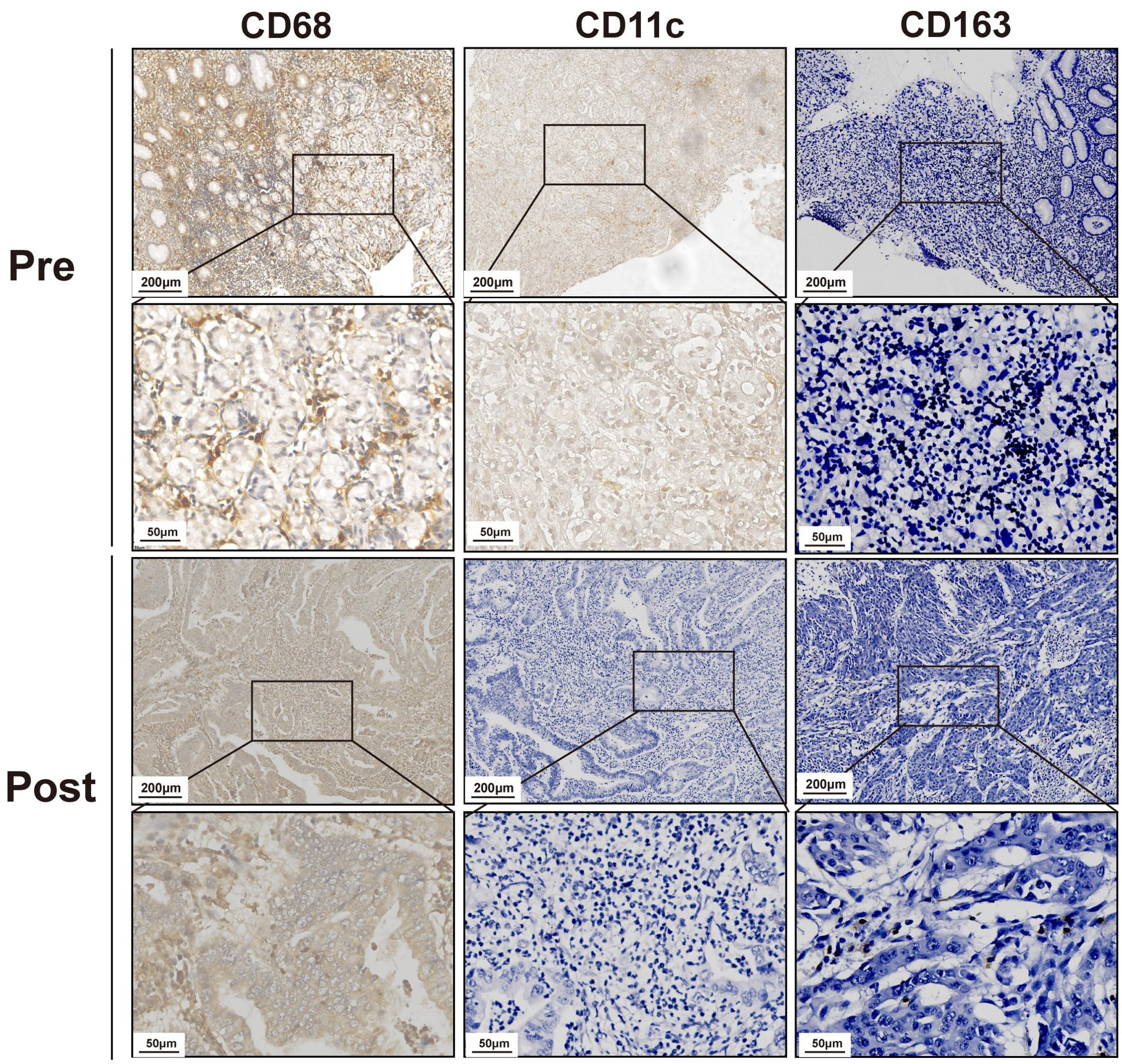

Figure S4D. Changes in macrophages in the non-response group before and after NACT for GC.
